# Germline variants in patients diagnosed with pediatric soft tissue sarcoma

**DOI:** 10.2340/1651-226X.2024.40730

**Published:** 2024-07-22

**Authors:** Synnøve Yndestad, Hans Kristian Haugland, Dorota Goplen, Dorota Wojcik, Stian Knappskog, Per Eystein Lønning

**Affiliations:** a K.G. Jebsen Center for Genome-Directed Cancer Therapy, Department of Clinical Science, University of Bergen, Bergen, Norway; b Department of Oncology, Haukeland University Hospital, Bergen, Norway; c Department of Pathology, Haukeland University Hospital, Bergen, Norway; d Department of Pediatrics, Haukeland University Hospital, Bergen, Norway

**Keywords:** Soft tissue sarcoma, pathogenic germline variants, hereditary, *MYO3A*, *MYO5B*, *CHD1L*

## Abstract

**Background:**

While soft tissue sarcomas affect younger patients, few studies have assessed the distribution of underlying pathogenic germline variants.

**Patients and methods:**

We retrospectively identified all pediatric and young adult patients (0–22 years) at Haukeland University Hospital, Norway (1981–2019), through clinical and pathological records. We identified *n* = 46 eligible patients. From these 46 patients, adequate material representing normal tissue was available for *n* = 41 cases (*n* = 24 diagnosed with rhabdomyosarcoma, 9 with synovial sarcomas, 2 with Ewing sarcomas, and 6 without further classification), with matching tumor tissue for *n* = 40. Normal tissue samples were analyzed for germline pathogenic variants (PVs) by targeted sequencing of 360 cancer genes.

**Results:**

Out of the 41 analyzed cases, we found PVs or likely PVs in 7 (17%). These variants were found in *TP53*, *MUTYH*, *FANCC*, *DICER1*, *FANCA, MYO3A*, and *MYO5B*. Supporting the causality of these PVs, four cases revealed loss of heterozygosity (LOH) of the wild-type allele in the tumor tissue, one patient with a PV in *DICER1* had a second somatic variant in *DICER1,* and a patient with a PV in *TP53* had the altered allele amplified in the tumor. For three out of five with available family history, a history of other cancers in relatives was recorded. Among genes with variants of uncertain significance, *CHD1L* was of particular interest, revealing a stop-gain and a missense variant.

**Interpretation:**

A high fraction of young patients with soft tissue sarcoma harbor PVs. Among the genes affected, we substantiate a potential role of *MYO5B* and propose a potential role for *MYO3A*.

## Graphical abstract

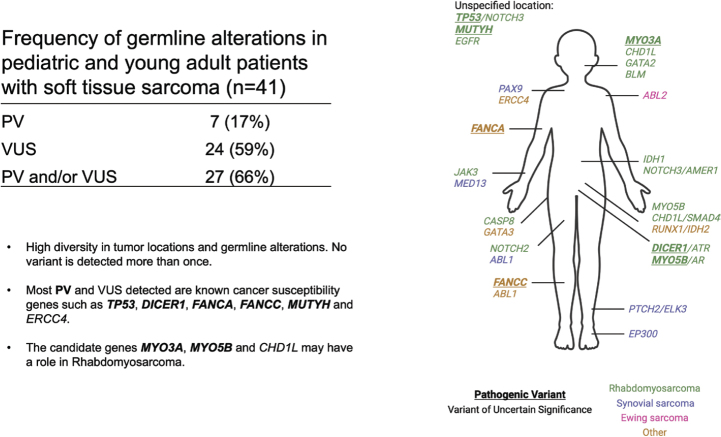

## Introduction

Sarcoma is a highly diverse group of cancers originating from mesenchymal cells in the bones or soft tissues. While sarcomas make up less than 1% of all adult solid malignant cancers, they account for more than 20% of solid malignant cancers in the pediatric population [1]. Some pediatric solid tumors, like retinoblastomas and Wilms tumors, are strongly linked to defined monogenetic germline variants but for the majority of pediatric cancers, including sarcomas, the contribution of germline alterations is less well defined [2].

Rhabdomyosarcoma (RMS) is the soft tissue sarcoma most frequently diagnosed in childhood [3]. There are two major subtypes. Embryonal RMSs accounting for about 70% of cases has a peak incidence around 2 to 4 years of age. The alveolar subtype, characterized by frequent translocations between *PAX3* or *PAX7* and *FOXO1* genes, is diagnosed in children and young adults up to 20 years of age with no distinct age peak [4, 5]. While RMS has been associated with rare cancer-disposition syndromes such as the Li–Fraumeni syndrome and neurofibromatosis type-1, these syndromes account for a limited number of cases [6]. Recent studies have estimated the fraction of RMSs associated with pathogenic germline variants to be less than 10% [7, 8].

Searching for potential cancer-predisposing germline variants in childhood cancers provides several challenges. These tumors are extremely rare; thus, even assuming a relative odds ratio of > 50, similar to *BRCA1* and breast cancer risk [9], the likelihood of detecting several cases of pediatric/adolescent malignancies in a family is small. Then, before the time of modern treatment, most children diagnosed with a pediatric solid tumor died before reaching age of reproduction, limiting the possibility of identifying germline aberrations in familial linkage studies. A remaining feasible strategy is to assess germline status in individual patients and, after detection of potentially pathogenic variants, identify additional indications of causality, for example, LOH in tumors.

Conflicting evidence has linked congenital malformations to risk of pediatric cancers [10–14]. During screening of potential germline variants in a 20-year-old man with RMS located in the prostate, we identified a germline variant in the *CHD1L* gene (amplified in liver cancer 1; *ALC1*), encoding a chromodomain helicase in which mutations have been associated with urinary tract anomalies [15, 16]. Triggered by this finding, we initiated the present study to screen for germline variants across archive samples from young patients diagnosed with soft tissue sarcomas to potentially detect novel risk variants for these malignancies.

## Patients and methods

### Patients

Pediatric and young adult (0–22 years) patients, treated for soft tissue sarcomas, in our institution between year 1981–2019 was retrospectively identified through hospital (clinical and pathological) records. We identified *n* = 46 eligible patients with pathological confirmed diagnosis of soft tissue sarcoma and availability biomaterial in our diagnostic biobank. From the 46 patients, adequate material representing normal tissue was available for *n* = 41 cases, with matching tumor tissue for *n* = 40.

### Ethics

The protocol was approved by the Regional Ethical Committee of Western Norway (REK vest 50564.2019).

### DNA extraction, library prep, and sequencing

Normal and tumor tissue samples were collected from FFPE blocks by extracting 0.8–1 mM core (5 mg tissue). DNA was isolated by adaptive focused acoustics (AFA)-based extraction using the Covaris truXTRAC FFPE DNA kit (Woburn, MA, USA) as previously described (Supplementary Information and [17]).

DNA (normal and tumor samples) was analyzed by targeted massive parallel sequencing of a 360 cancer gene panel, as previously described [18].

### Classification of germline variants

After sequencing and initial processing using the local run manager software (Illumina MiSeq instrument), variants in known cancer predisposition genes were classified according to the ACMG criteria [19] using the ‘Cancer Predisposition Sequencing Reporter’ (CPSR) module within the python package ‘The Personal Cancer Genome Reporter’ (PCGR) v 1.0.3 [20] or by hard filtering and manual curation for genes not covered by PCGR (see Supplementary Information).

### Somatic variant calling

Alignment was performed using MiSeq reporter against UCSC hg19, and functional annotation was performed by Annovar [21]. For the matched tumor-normal pairs, mutations and small indels were called by CaVEman [22] and Pindel [23], respectively. Copy number analysis on matched tumor and normal tissue was performed using FACET (https://github.com/mskcc/facets) [24].

## Results

For the period between 1981 and 2019, 46 pediatric, teenage, and young adult patients treated for soft tissue sarcoma at Haukeland University Hospital were identified. The age range was 0–22 years; 22 cases were female while 24 were male. Adequate normal tissue was available for 41 cases, and matched tumor-normal pairs was available for 40 cases (Table 1, Supplementary Data 2).

**Table 1:** Patient and tumor characteristics of included soft tissue sarcoma cases.

Patient and tumor characteristics	*N* = 41
**Histological diagnosis,** *n* (%)	
Rhabdomyosarcoma	24 (59%)
Synovial sarcoma	9 (22%)
Ewing sarcoma	2 (4.9%)
Other	6 (15%)
**Tumor location,** *n* (%)	
Abdominal	2 (5.6%)
Extremities	10 (28%)
Genitourinary	4 (11%)
Gynecologic	1 (2.8%)
Head and neck	8 (22%)
Pelvic	2 (5.6%)
Trunk	9 (25%)
Unknown	5
**Age at diagnosis**	
Mean (Median)	10.4 (11.0)
Range	0.0, 21.0
**Sex,** *n* (%	
Female	20 (49%)
Male	21 (51%)
**Diagnosis year**	
Mean (Median)	2007 (2010)
Range	1981, 2019
Unknown	1
**Matched germline and tumor,** *n* (%)	
Germline	1 (2.4%)
Germline/Tumor	40 (98%)

As for histopathology, 24 tumors (59%) were classified as RMSs, 9 as synovial sarcoma, two Ewing sarcomas affecting soft tissue, while the remaining 6 were sarcomas without further classification (Table 1). Genetic alterations in the tumors are summarized in Supplementary Figure 2. The most frequently somatically mutated genes were *NRAS*, *FGFR4*, and *CTNNB1,* which are all commonly mutated in RMSs [25].

### Germline pathogenic variants

**Figure 1:**
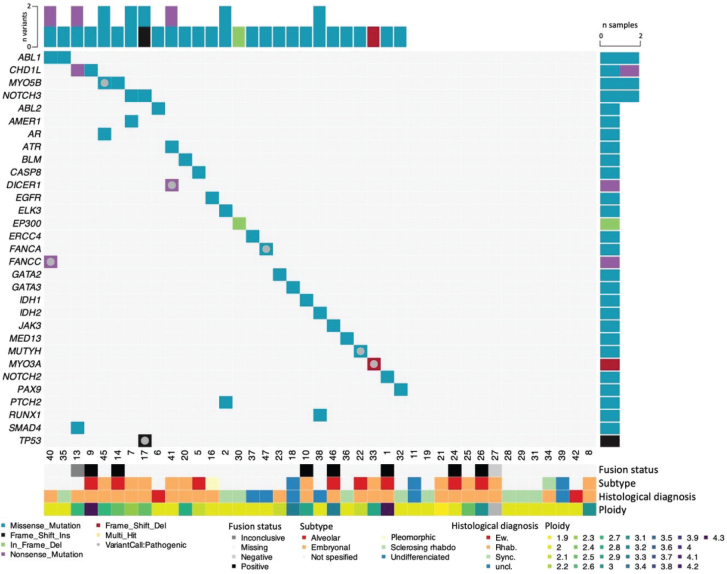
Germline variants in young patients with soft tissue sarcoma. Oncoplot presenting germline pathogenic variants and variants of uncertain significance (VUS) detected in selected genes (rows) in soft tissue sarcoma patients (columns). Pathogenic and likely pathogenic variants are indicated by a grey circle. The remaining variants are classified as VUS.

Out of the 41 analyzed cases, we found germline pathogenic or likely pathogenic germline variants (PV) in 7 cases (17%; Figure 1; Table 2). Among the detected variants, 5 were in known cancer predisposition genes (*TP53*, *MUTYH*, *FANCC*, *DICER1*, and *FANCA*), while one patient harbored a variant in *MYO3A,* and another one in *MYO5B*. Both the latter genes belong to the myosin family, and functional loss of *MYO3A* and *MYO5B* disrupts cell polarity and causes malformation of cochlea hairs and microvillus leading to deafness and microvillus inclusion disease, respectively [26, 27].

**Table 2:** Germline pathogenic variants and variants of uncertain significance in pediatric soft tissue sarcoma.

Sample ID	Gene	Chr.	Position	Ref.	Alt.	Variant Class	AA Change	Variant Call	VAF blood	VAF tumor	Variant CNA^*1*^	Cancer type	Subtype	Fusion status	Primary site	Age	Sex	Familial cancer^*2*^
017n	*TP53*	chr17	7579418	G	GA	Frame shift	p.S90fs	Pathogenic	0.4000	0.5965	Amp	Rhab.	Embryonal	NA	NA	7	Male	Y
041n	*DICER1*	chr14	95578563	G	A	Nonsense	p.R688X	Pathogenic	0.4850	0.4897	NA3	Rhab.	Embryonal	NA	Gynecologic	15	Female	Y
022n	*MUTYH*	chr1	45799108	G	A	Missense	p.R106W	Likely pathogenic	0.4302	0.9256	LOH	Rhab.	Alveolar	NA	NA	7	Male	N
033n	*MYO3A*	chr10	26463065	A	-	Frame shift	p.Q1291fs	Likely pathogenic	0.5196	0.7045	LOH	Rhab.	Embryonal	NA	Head and neck	13	Female	Unknown
040n	*FANCC*	chr9	97864024	G	A	Nonsense	p.R548X	Likely pathogenic	0.2941	0.5096	NA	uncl.	Unknown	NA	Extremities	0	Male	Unknown
045n	*MYO5B*	chr18	47500736	C	A	Missense	p.V436F	Likely pathogenic	0.5043	0.5750	LOH	Rhab.	Embryonal	NA	Pelvic	1	Female	N
047n	*FANCA*	chr16	89813256	T	C	Missense	p.T1131A	Likely pathogenic	0.4706	0.5455	LOH	uncl.	Unknown	NA	Extremities	19	Male	Y
001n	*NOTCH2*	chr1	120497768	C	T	Missense	p.R705H	VUS	0.3947	0.4310	No	Rhab.	Alveolar	positive	Extremities	7	Male	NA
002n	*PTCH2*	chr1	45297405	T	C	Missense	p.K197R	VUS	0.4865	0.4360	No	Sync.	Unknown	NA	Extremities	6	Female	NA
002n	*ELK3*	chr12	96653576	G	A	Missense	p.S357N	VUS	0.5000	0.5031	No	Sync.	Unknown	NA	Extremities	6	Female	NA
005n	*CASP8*	chr2	202149811	G	C	Missense	p.A418P	VUS	0.4769	0.3827	No	Rhab.	Alveolar	NA	Trunk	9	Female	NA
006n	*ABL2*	chr1	179090902	G	A	Missense	p.A263V	VUS	0.5102	0.5294	No	Ew.	Unknown	NA	Trunk	17	Male	NA
007n	*NOTCH3*	chr19	15276251	G	A	Missense	p.R1915C	VUS	0.5085	0.5366	No	Rhab.	Embryonal	NA	Abdominal	2	Female	NA
007n	*AMER1*	chrX	63412159	G	C	Missense	p.D336E	VUS	0.4125	0.4974	NA	Rhab.	Embryonal	NA	Abdominal	2	Female	NA
009n	*CHD1L*	chr1	146743877	G	A	Missense	p.R402K	VUS	0.3784	0.1036	No	Rhab.	Alveolar	PAX3	Head and neck	12	Male	NA
010n	*IDH1*	chr2	209108301	T	C	Missense	p.Y183C	VUS	0.4519	0.3281	No	Rhab.	Embryonal	PAX3	Abdominal	6	Female	NA
013n	*SMAD4*	chr18	48575164	G	T	Missense	p.D120Y	VUS	0.4667	0.4403	NA	Rhab.	Unknown	inconclusive	Genitourinary	20	Male	Y
013n	*CHD1L*	chr1	146766153	C	T	Nonsense	p.R857X	VUS	0.4652	0.4138	No	Rhab.	Unknown	inconclusive	Genitourinary	20	Male	Y
014n	*MYO5B*	chr18	47431169	G	A	Missense	p.A815V	VUS	0.5083	0.5446	NA	Rhab.	Alveolar	PAX7	Genitourinary	3	Female	NA
016n	*EGFR*	chr7	55259485	C	T	Missense	p.P848L	VUS	0.5507	0.4032	No	Rhab.	Pleomorph	NA	NA	12	Male	N
017n	*NOTCH3*	chr19	15302615	C	G	Missense	p.G248A	VUS	0.4837	0.5217	No	Rhab.	Embryonal	NA	NA	7	Male	Y
018n	*GATA3*	chr10	8097700	C	A	Missense	p.H28N	VUS	0.4854	0.9239	LOH	uncl.	Undifferentiated	NA	Trunk	11	Female	NA
020n	*BLM*	chr15	91312417	C	A	Missense	p.L788I	VUS	0.4300	0.4387	NA	Rhab.	Embryonal	NA	Head and neck	4	Male	NA
023n	*GATA2*	chr3	128205754	G	C	Missense	p.P41A	VUS	0.4367	0.4789	NA	Rhab.	Unknown	NA	Head and neck	14	Male	NA
030n	*EP300*	chr22	41574340	AACC AGTT CCAGC	A	In frame del	p.N2209_ Q2213delinsK	VUS	0.2961	0.2110	NA	Sync.	Unknown	NA	Extremities	20	Female	NA
032n	*PAX9*	chr14	37132699	T	A	Missense	p.I201N	VUS	0.5526	0.4936	NA	Sync.	Unknown	NA	Trunk	19	Male	NA
035n	*ABL1*	chr9	133730199	C	T	Missense	p.R89W	VUS	0.3929	0.4177	No	Sync.	Unknown	NA	Extremities	20	Female	NA
036n	*MED13*	chr17	60023885	G	A	Missense	p.P2157S	VUS	0.4985	0.5111	No	Sync.	Unknown	NA	Extremities	5	Female	NA
037n	*ERCC4*	chr16	14041606	T	C	Missense	p.L718P	VUS	0.4375	0.5128	NA	uncl.	Unknown	NA	Trunk	2	Female	NA
038n	*RUNX1*	chr21	36259336	A	T	Missense	p.M52K	VUS	0.4804	0.4935	LOH	uncl.	Unknown	NA	Genitourinary	2	Male	NA
038n	*IDH2*	chr15	90630756	A	G	Missense	p.W244R	VUS	0.4561	0.4600	No	uncl.	Unknown	NA	Genitourinary	2	Male	NA
040n	*ABL1*	chr9	133738171	C	T	Missense	p.R191C	VUS	0.4211	0.4783	NA	uncl.	Unknown	NA	Extremities	0	Male	Unknown
041n	*ATR*	chr3	142231224	C	A	Missense	p.S1577I	VUS	0.4750	0.4605	No	Rhab.	Embryonal	NA	Gynecologic	15	Female	Y
045n	*AR*	chrX	66766412	C	T	Missense	p.A475V	VUS	0.6418	0.4286	No	Rhab.	Embryonal	NA	Pelvic	1	Female	N
046n	*JAK3*	chr19	17940964	G	T	Missense	p.L1054M	VUS	0.5789	0.7353	LOH	Rhab.	Alveolar	positive	Extremities	2	Male	Unknown

VUS: variants of uncertain significance, VAF: variant allele frequency, CNA: copy number alteration

1 NA = Not assessed, too few SNV in area to assess accurate CNA with FACET.

2 NA = Not available. Unknown = Patient journal examined, but no relevant information found.

^*3*^Patient 41 also harbored a somatic mutation in DICER1.

Regarding sarcoma subtypes, 6 of the 7 PVs were found in patients with RMS, while the last was found in patients with unclassified sarcoma (Supplementary Table 1). No pathogenic variants were discovered among individuals diagnosed with synovial sarcomas (*n* = 9) or Ewing sarcomas (*n* = 2). The presence of PVs was not biased with respect to gender, with 4 out of 7 found in males and 3 found in females. The median age of onset was 7 years among the patients with a PV, and 11 years for those with no PV detected, but this difference did not reach statistical significance (*p* > 0.4). The location of primary tumors in patients with a PV was diverse and did not differ from the primary sites of the tumors in patients with no PV (Table 2).

Out of the alterations classified here as PVs, the *MUTYH* p.R106W (dbSNP: rs765123255), *FANCC* p.R548X (dbSNP: rs104886457), *DICER1* p.R688X (dbSNP: rs886037684), and *FANCA* p.T1131A (dbSNP: rs574034197) have previously been reported as pathogenic in ClinVar. Although other nucleotide changes causing *TP53* S90fs have been reported the presently detected variant causing *TP53* S90fs as well as the *MYO3A* Q1291fs and *MYO5B* V436F variants have not been reported in either the 1,000 genomes (1000g2014oct_all) or the gnomAD (v2.1.1) database.

Supporting the causality of the PVs with respect to tumorigenesis, 4 tumors revealed LOH with loss of the wild-type allele in the tumor tissue (Table 2; Supplementary Figure 3). For the patient with a PV in *DICER1,* LOH status was not assessable due to too few SNPs in the region. A second somatic variant in *DICER1* was, however, found in the tumor (Figure 1, Supplementary Figures 2 and 3). Further, the patient with a PV in *TP53* had the altered allele amplified in the tumor, while CNA status was unavailable for the patient with a *FANCC* variant.

Out of the 7 genes in which we detected PVs, 4 (*TP53*, *DICER1, FANCC,* and *FANCA*), have previously been reported associated with elevated risk of RMSs. The primary tumor type for those affected by PVs in these genes, in the present study, were embryonal RMS (*TP53*, *DICER1*), rhabdomyofibrosarcoma (*FANCC*), and osteosarcoma (*FANCA*). Interestingly, the tumors in patients with the novel variants in *MYO3A* and *MYO3B*, as well as *MUTYH,* were all RMSs. These genes have not previously been shown to be associated with elevated risk of either RMSs, Ewing sarcoma or soft tissue sarcomas in general. It should be noted that two patients in our cohort had somatic mutations in *MYO3A* and *MYO3B* (patients 17 and 33, respectively), further indicating a role for these genes in sarcoma (Supplementary Figure 2 and Supplementary Data 3).

Of the 7 patients with a PV, information about family history of cancer was available for 5. Out of these, 3 had a family history of cancer: the patients carrying the *TP53*, *FANCA* and *DICER* variants (Table 2; further detail in Supplementary Information).

### Variants of uncertain significance

In addition to the pathogenic and likely pathogenic variants, we performed an in-depth assessment of variants of uncertain significance (VUS), in order to potentially reveal previously unknown mechanisms of early onset soft tissue sarcoma. We identified a total of 28 VUS (Figure 1; Table 2). Overall, the 41 patients harbored on average 0.7 VUS with few recurrent genes (Table 2). Among the patients harboring either a pathogenic variant or a VUS, the patients were diagnosed at a younger age (median 7 vs. 13 years) although this difference was not statistically significant.

## Discussion

In the present study, we found 7 out of 41 young patients diagnosed with soft tissue sarcomas to harbor pathogenic or likely pathogenic germline variants.

In 5 out of the 7 cases with PVs, the variants were found in well-established cancer risk genes (*TP53*, *MUTYH*, *FANCC*, *DICER1*, and *FANCA*). Although the number of observations is small, it is interesting to note that four of these genes have critical roles in DNA repair, similar to what is found in other reports on early onset sarcoma [28, 29].

In addition to PVs in these five genes, we detected germline PVs in *MYO3A* and *MYO5B,* both involved in elongation of actin in stereocilia tips and epithelial polarization [26, 30]. Some potentially damaging germline variants in these two genes have been reported previously in different other cancer types including CNS tumors, neuroblastoma (*MYO3A*), and single cases of osteosarcomas and RMSs (*MYO5B*) [31]. A potential role of these genes to RMS evolution is further supported by our finding of somatic variants in both *MYO3A* and *MYO5B* in two different RMSs. In a study reporting germline PVs, among 1120 pediatric cancer patients [32] neither *MYO3A*, *MYO3B,* or *CHD1L* were covered. Thus, our present findings represent novel information about the potential role of these genes in sarcoma. Pending further validation, this may have clinical implications with respect to how variants in these genes are considered in the setting of genetic counselling.

We found VUS in several genes, where *CHD1L* is of particular interest. In addition to the stop-gain mutation in patient no. 013, which initiated the present study, a germline missense VUS, was detected in patient 009. Further, one patient (no.025) revealed a somatic *CHD1L* mutation in the tumor). Interestingly, a previous study [31] found *CHD1L* germline alterations among three pediatric patients diagnosed with neuroblastoma, retinoblast-oma, and a Wilms tumor, respectively.

While the pathogenic or likely pathogenic variants affected canonical cancer predisposition genes, the VUSes affected a broader repertoire of genes. To seek insight into the biology of variants observed in early onset sarcoma, we assessed the genes affected by VUSes for their association with syndromes other than cancer. Several of the genes were associated with developmental/birth defects and/or with defects in the same organ system in which the sarcomas of our cases originated. PVs in *CHD1L* have also been linked to congenital anomaly of kidneys and the urinary tract [15]. Another important example is *MYO3A* for which germline mutations are associated with deafness. Patient no 33, harbouring a pathogenic variant in *MYO3A*, was diagnosed with a RMS located in the soft palate. Although these observations should be seen as anecdotal observations, we believe they warrant further investigations into the potential link between genetic alterations in developmental defect syndromes and sarcomas.

In conclusion, we find a relatively high fraction of young patients with soft tissue sarcoma to harbor pathogenic or likely pathogenic germline variants. Among the genes affected by PVs, we substantiate the potential role of *MYO5B* and propose a potential role for *MYO3A*. In addition, our data suggest that *CDH1L* may be a candidate for further investigation with respect to risk of soft tissue sarcoma.

## Author contributions

SY: Performed data analysis and interpreted results wrote the manuscript.

DG: Reviewed and identified cases provided clinical information.

DW: Reviewed and identified cases provided clinical information.

HKH: Reviewed and identified cases performed pathological review and provided clinical information.

SK: Interpreted results, provided funding, wrote the manuscript.

PEL: Conceived the study, interpreted results, provided funding, wrote the manuscript.

All authors: Approved the final version of the manuscript.

## Supplementary Material

Germline variants in patients diagnosed with pediatric soft tissue sarcoma

Germline variants in patients diagnosed with pediatric soft tissue sarcoma

Germline variants in patients diagnosed with pediatric soft tissue sarcoma

Germline variants in patients diagnosed with pediatric soft tissue sarcoma

## Data Availability

Filtered data are available in Supplementary Data 1–4. Raw data are collected, stored, and disseminated according to institutional guidelines. After publication and on formal request, raw data, including deidentified individual participant data, may be shared according to institutional procedures. Requests are via a standard pro forma describing the nature of the proposed research and extent of data requirements. Data recipients are required to enter a formal data sharing agreement, which describes the conditions for release and requirements for data transfer, storage, archiving, publication, and intellectual property. Requests are reviewed by the study team (authors of the present paper) in terms of scientific merit and ethical considerations.
