# Supplementary material for: Germline variants in patients diagnosed with pediatric soft tissue sarcoma
Source: Acta Oncol. 2024 Jul 22;63:40730. doi: 10.2340/1651-226X.2024.40730 (PMC11332449; doi:10.2340/1651-226X.2024.40730)

Supplementary material has been published as submitted. It has not been copyedited, or typeset by Acta Oncologica

**Supplementary figure 1.** Gene panels. Venn diagram illustrating the level of overlap between the analysed 360-gen panel and the Cancer predisposition super panel (CPRS).

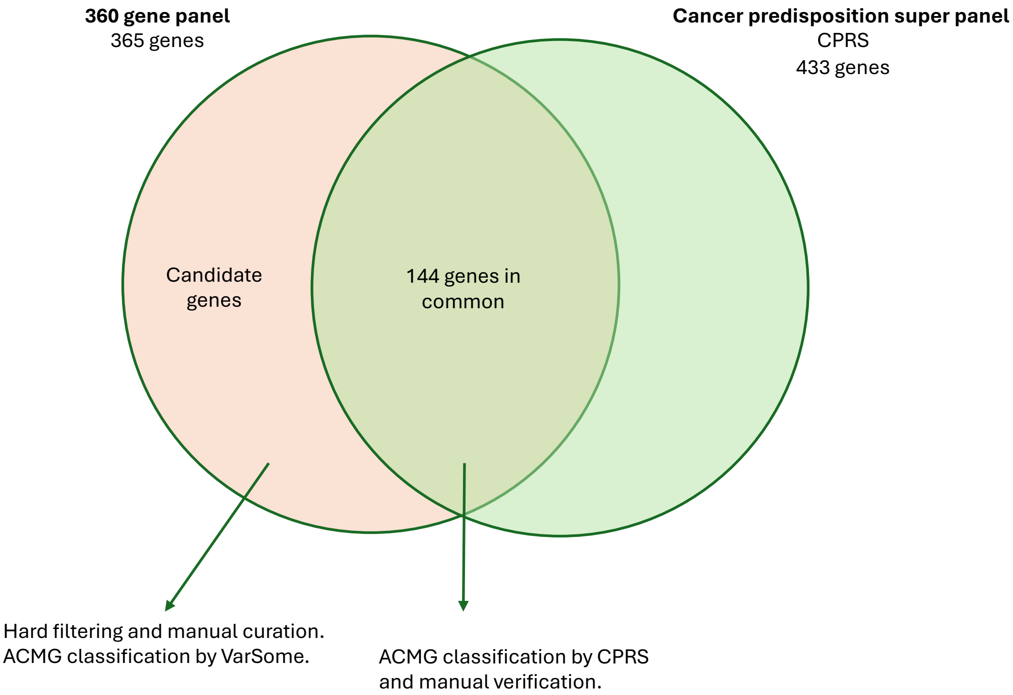

**Supplementary figure 2. (A)** Summary of somatic variant classification, type and SNV class in soft tissue sarcoma samples (pooled data from 41 patients). **(B)** Overview of somatic transitions and transversions in soft tissue sarcoma samples (pooled data from 41 patients). **(C)** Somatic variants in soft tissue sarcoma. Oncoplot presenting somatic mutations detected in selected genes (rows) in soft tissue sarcoma samples (columns).

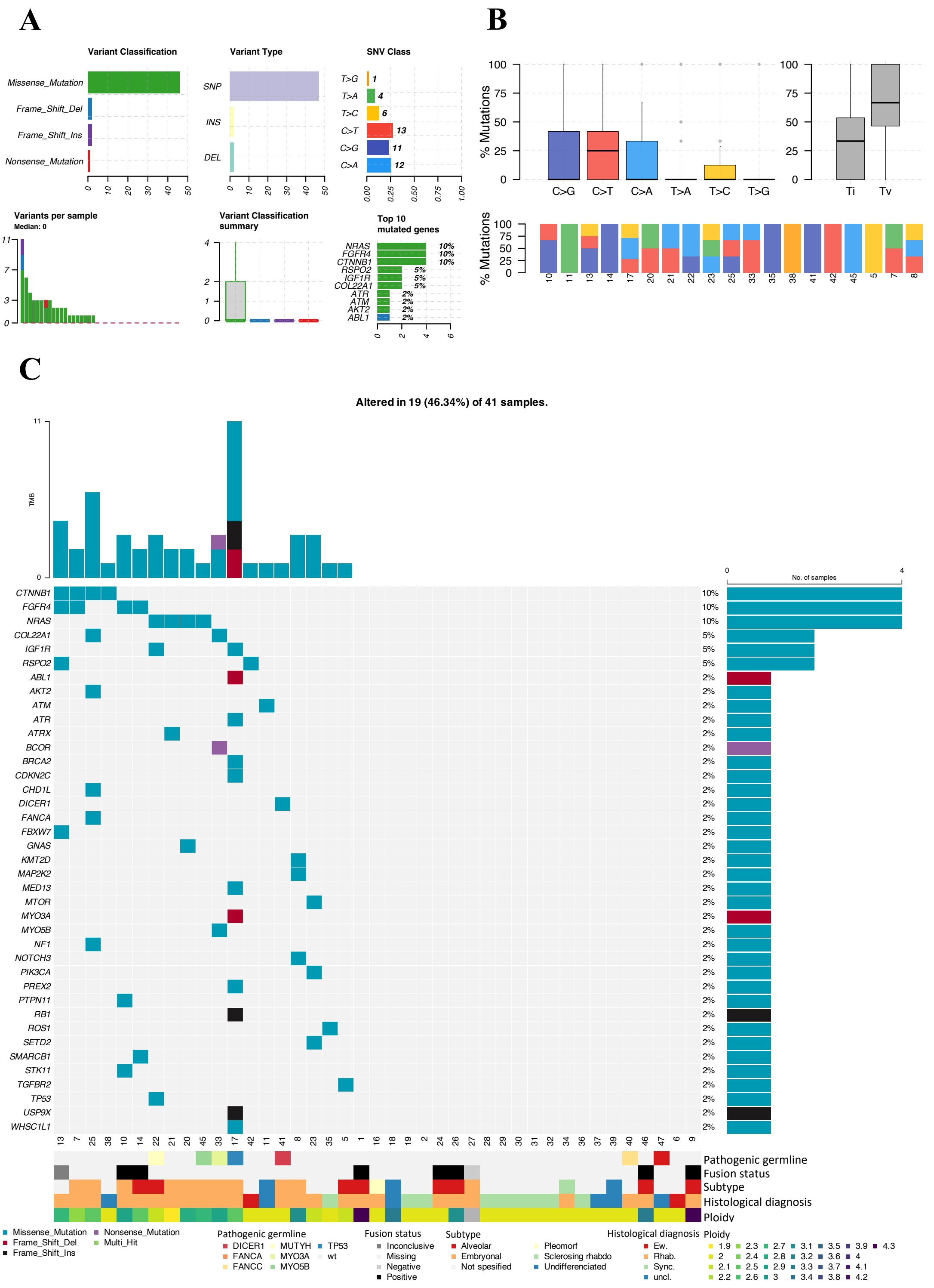

**Supplementary figure 3.** Second hits. Copy number alterations (CNA) as called by the FACTS algorithm, based on sequencing of a targeted 360 gen panel. Copy number status for genes affected by pathogenic or likely pathogenic germline variants are indicated of the respective patients. For patient no.041 the CNA status was not possible to determine for chromosome 14, but this patient had a second hit in *DICER1* in the form of a somatic mutation.

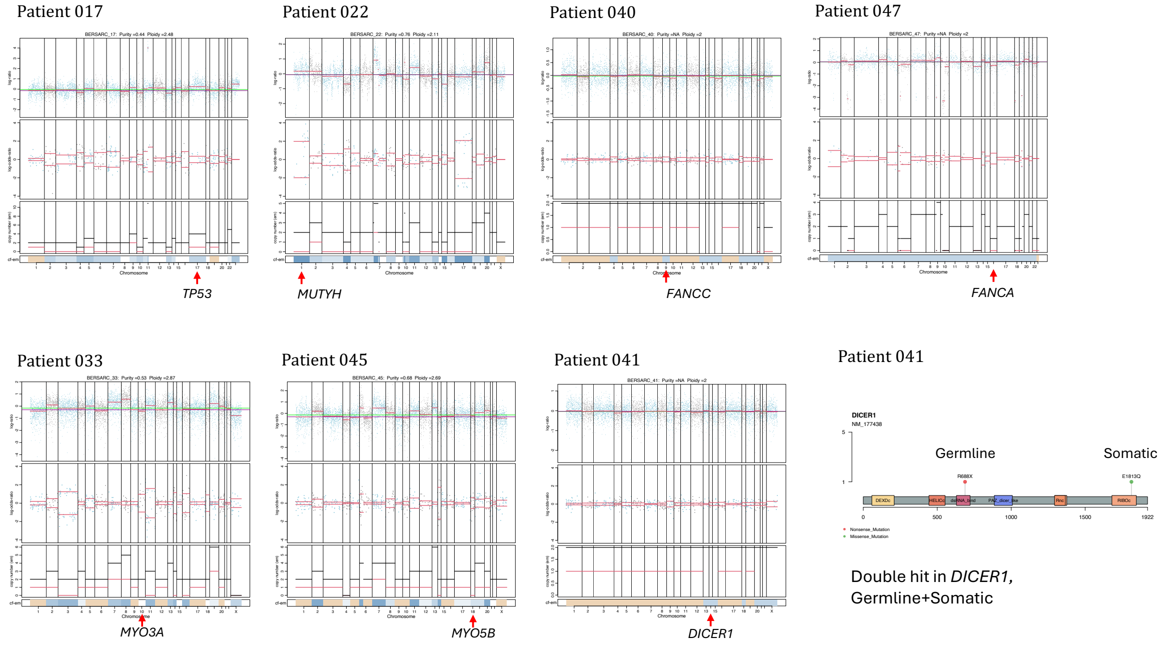

Supplement: Germline variants in patients diagnosed with pediatric soft tissue sarcoma [file AO-63-40730-s2.pdf]
