# Supplementary material for: Germline variants in patients diagnosed with pediatric soft tissue sarcoma
Source: Acta Oncol. 2024 Jul 22;63:40730. doi: 10.2340/1651-226X.2024.40730 (PMC11332449; doi:10.2340/1651-226X.2024.40730)
Supplement: Germline variants in patients diagnosed with pediatric soft tissue sarcoma [file AO-63-40730-s3.pdf]

Characteristic	**Pathogenic/VUS**, N = 27[1]	**wt**, N = 14[1]	p-value[2]	**Pathogenic**, N = 7[1]	**wt**, N = 34[1]	p-value[3]	**VUS**, N = 24[1]	**wt**, N = 17[1]	p-value[2]
**Histological diagnosis**			0.8			0.4			>0.9
Rhabdomyosarcoma	17 (63%)	7 (50%)		6 (86%)	18 (53%)		15 (63%)	9 (53%)	
Synovial sarcoma	5 (19%)	4 (29%)		0 (0%)	9 (26%)		5 (21%)	4 (24%)	
Ewing sarcoma	1 (3.7%)	1 (7.1%)		0 (0%)	2 (5.9%)		1 (4.2%)	1 (5.9%)	
Other	4 (15%)	2 (14%)		1 (14%)	5 (15%)		3 (13%)	3 (18%)	
**Tumor location**			0.7			0.13			0.7
Abdominal	2 (8.3%)	0 (0%)		0 (0%)	2 (6.5%)		2 (9.1%)	0 (0%)	
Extremeties	8 (33%)	2 (17%)		2 (40%)	8 (26%)		7 (32%)	3 (21%)	
Genitourinary	3 (13%)	1 (8.3%)		0 (0%)	4 (13%)		3 (14%)	1 (7.1%)	
Gynecologic	1 (4.2%)	0 (0%)		1 (20%)	0 (0%)		1 (4.5%)	0 (0%)	
Head and neck	4 (17%)	4 (33%)		1 (20%)	7 (23%)		3 (14%)	5 (36%)	
Pelvic	1 (4.2%)	1 (8.3%)		1 (20%)	1 (3.2%)		1 (4.5%)	1 (7.1%)	
Trunk	5 (21%)	4 (33%)		0 (0%)	9 (29%)		5 (23%)	4 (29%)	
Unknown	3	2		2	3		2	3	
**Age at diagnosis**			0.2			0.5			0.10
Median (IQR)	7.0 (3.5, 14.5)	13.0 (10.0, 14.8)		7.0 (4.0, 14.0)	11.0 (5.3, 14.8)		7.0 (2.8, 14.3)	13.0 (10.0, 15.0)	
**Sex**			>0.9			>0.9			0.9
Female	13 (48%)	7 (50%)		3 (43%)	17 (50%)		12 (50%)	8 (47%)	
Male	14 (52%)	7 (50%)		4 (57%)	17 (50%)		12 (50%)	9 (53%)	

[1] n (%)

[2] Fisher's exact test; Wilcoxon rank sum test; Pearson's Chi-squared test

[3] Fisher's exact test; Wilcoxon rank sum test

VUS: variant of uncertain significance, wt: wild-type
