# Supplementary material for: Germline variants in patients diagnosed with pediatric soft tissue sarcoma
Source: Acta Oncol. 2024 Jul 22;63:40730. doi: 10.2340/1651-226X.2024.40730 (PMC11332449; doi:10.2340/1651-226X.2024.40730)
Supplement: Germline variants in patients diagnosed with pediatric soft tissue sarcoma [file AO-63-40730-s4.pdf]

# Supplementary Information

## 1.Suppementary Materials and Methods

**Patients**

Pediatric and young adult (0-22 years) patients, treated for soft tissue sarcomas, at Haukeland University Hospital between year 1981 and 2019 was retrospectively identified through hospital (clinical and pathological) records. We identified n=46 eligible patients. Inclusion criteria was a pathological confirmed diagnosis of soft tissue sarcoma, and availability in our diagnostic biobank of archival paraffin-embedded tumour and normal tissue for DNA extraction. From the 46 patients, adequate material representing normal tissue for DNA extraction was available for n=41 cases, with matching tumour tissue for n=40. None of the patients were excluded for reasons other than lacking biomaterial.

**DNA extraction, library preparation and sequencing**

Guided by a pathologist, normal and tumor tissue samples were collected from FFPE blocks for DNA extraction. To avoid the potential for contamination from tumor cells in samples representing normal tissue, samples were collected by extracting 0.8-1 mM cores from areas of interest (approximately 5 mg tissue per sample) applying a TMA-punch needles mounted in a Tissue Arrayer.

DNA was isolated using Adaptive Focused Acoustics (AFA)-based extraction using the Covaris truXTRAC FFPE DNA kit (Woburn, MA, USA) according to the protocol provided by Covaris as previously described [1]. In brief, we used a Covaris M220 Focused Ultrasonicator to perform AFA using protocol C. After removing paraffin, the tissue was rehydrated in 100µl processing buffer master mix containing 88µl of tissue and SDS mixed with 22 µl of proteinase K (EC.3.4.21.64, Product No. SRE0005, Sigma–Aldrich). To reverse the formaldehyde crosslinks, the homogenized tissue was digested overnight at 56°C followed by 1h incubation at 80°C. Columns from Covaris truXTRAC FFPE DNA kit was used to isolate DNA from the digested lysate, and DNA was eluted in 100µl of Covaris BE buffer. In order to repair some of the damage from formalin fixation and paraffin embedding, we used the DNA repair mix kit (NEBNext FFPE DNA kit, UK), according to the manufacturer’s instructions.

We performed targeted massive parallel sequencing using a 360 gene panel of cancer-related genes, as previously described [2]. The SureSelect XT protocol (Agilent, US) is a hybrid capture-based target enrichment procedure optimized for use of 1 µg of DNA per sample with SureSelectXT Capture Library baits (Design ID #0764931, Agilent). The libraries were sequenced on an Illumina MiSeq instrument, aiming at a mean target coverage of 200x per sample.

**Classification of germline variants:**

After sequencing and initial processing using the local run manager software on an Illumina MiSeq instrument (including mutation calling with removal of low VAF-variants), variants in known cancer predisposition genes was classified according to the ACMG criteria [3] using the "Cancer Predisposition Sequencing Reporter" (CPSR) module within the python package "The Personal Cancer Genome Reporter" (PCGR) v 1.0.3 [4]. CPRS for automated ACMG classification was run in a conda environment, (using maf_upper_threshold = 0.1 and panel_id = 0 settings) with the CPRS super panel (panel 0). All variants flagged as Pathogenic or VUS by CPRS (n=154 variants), were manually re-evaluated in ClinVar. In ClinVar, if the variant had stronger evidence for benign status than for uncertain, the variant was removed. This assessment was based on the number of submissions, the strength of the submissions evidence and the presence of functional studies. This manual curation removed 52 variants, leaving 102 potential variants from the CPRS classification in the super panel.

The applied targeted gene panel of 360 genes consists of a mix of cancer related genes, originally selected based on frequencies of somatic mutations. In the context of cancer predisposition, around half of the genes are known cancer predisposition genes, while the remaining may be considered candidate genes. (Supplementary figure 1; Supplementary data 1). The CPRS super-panel, Panel 0, contains 433 curated genes relevant for cancer predisposition, out of which 144 are overlapping with the current 360 gene panel.

The remaining candidate genes were assessed for potential pathogenic variants manually using hard filtering. Germline variants were filtered on quality by keeping only variants with Filter = Pass, and Quality = 100. Genomic SuperDubs was removed. Variants in exonic regions was kept, while variants with a population frequency >= 1% in the population database of 1000 genomes (1000g2014oct_all and 1000g2014oct_eur) was removed. Further, the gnomAD database (https://gnomad.broadinstitute.org) was used, and all variants with population frequency >= 1% in the combined exome and genome database for European (non-Finnish) and/or Swedish population was removed. Synonymous variants and variants with low VAF (<0.25) in normal tissue was removed.

Further, Combined Annotation Dependent Depletion (CADD; https://cadd.gs.washington.edu), The Rare Exome Variant Ensemble Learner (REVEL) and Google DeepMinds AlphaMissense [5] were used to predict pathogenicity of SNVs (see Supplementary methods for details). All variants from both gene sets with a CADD-phred score ≥10, REVEL ≥0.5 and/or classified as Pathogenic by AlphaMissense were kept. All remaining SNV and all indel variants were then classified according to ACMG criteria by VarSome [6] and ClinVar annotations.

**Somatic variant calling:**

Alignment was performed using MiSeq reporter against UCSC hg19, and functional annotation was performed by Annovar [7]. For the matched tumor-normal pairs, mutations and small indels were called by CaVEman [8] and Pindel [9], respectively. Copy number analysis on matched tumor and normal tissue was performed using FACET (https://github.com/mskcc/facets) [10].

**Classification of gene and phenotype associations:**

Associations of hereditary conditions within genes was examined using ClinVar, the Online Catalog of Human Genes and Genetic Disorders (OMIM), panelApp (https://panelapp.genomicsengland.co.uk), and DatabasE of genomiC varIation and Phenotype in Humans using Ensembl Resources (DECIPHER, https://www.deciphergenomics.org).

## 2.Suppementary Results

**Family history**

Of the 7 patients with a PV, information about family history of cancer was available for 5. Out of these, 3 had a family history of cancer (Table 2). Patient 017 harboring a *TP53* frameshift variant (p.S90f) was diagnosed at 7 years of age and had a mother suffering from bilateral breast cancer at a young age. Case 047 was a carrier of the *FANCA* p.T1131A variant and his mother died from breast cancer at an early age (38y). Patient No 041 was found to carry a nonsense mutation in *DICER1* (p.R668X) and had a non-first degree relative with breast cancer. Two cases did not have a family history (022; carrying a *MUTYH* variant and 045; carrying a *MYO5B*), while the family history was unknown for two of the patients with PV (033 and 040, with variants in *MYO3A* and *FANCC*, respectively).

**Supplemental references**


1. Venizelos A, Elvebakken H, Perren A, Nikolaienko O, Deng W, Lothe IMB, et al. The molecular characteristics of high-grade gastroenteropancreatic neuroendocrine neoplasms. Endocrine-related cancer. 2022;29(1):1-14. doi: 10.1530/erc-21-0152.
2. Yates LR, Gerstung M, Knappskog S, Desmedt C, Gundem G, Van Loo P, et al. Subclonal diversification of primary breast cancer revealed by multiregion sequencing. Nature Medicine. 2015;21(7):751-+. doi: 10.1038/nm.3886.
3. Richards S, Aziz N, Bale S, Bick D, Das S, Gastier-Foster J, et al. Standards and guidelines for the interpretation of sequence variants: a joint consensus recommendation of the American College of Medical Genetics and Genomics and the Association for Molecular Pathology. Genetics in Medicine. 2015;17(5):405-24. doi: 10.1038/gim.2015.30.
4. Nakken S, Saveliev V, Hofmann O, Moller P, Myklebost O, Hovig E. Cancer Predisposition Sequencing Reporter (CPSR): A flexible variant report engine for high-throughput germline screening in cancer. International Journal of Cancer. 2021;149(11):1955-60. doi: 10.1002/ijc.33749.
5. Cheng J, Novati G, Pan J, Bycroft C, Zemgulyte A, Applebaum T, et al. Accurate proteome-wide missense variant effect prediction with AlphaMissense. Science. 2023;381(6664):eadg7492. doi: 10.1126/science.adg7492.
6. Kopanos C, Tsiolkas V, Kouris A, Chapple CE, Albarca Aguilera M, Meyer R, et al. VarSome: the human genomic variant search engine. Bioinformatics. 2019;35(11):1978-80. doi: 10.1093/bioinformatics/bty897.
7. Yang H, Wang K. Genomic variant annotation and prioritization with ANNOVAR and wANNOVAR. Nature Protocols. 2015;10(10):1556-66. doi: 10.1038/nprot.2015.105.
8. Jones D, Raine KM, Davies H, Tarpey PS, Butler AP, Teague JW, et al. cgpCaVEManWrapper: Simple Execution of CaVEMan in Order to Detect Somatic Single Nucleotide Variants in NGS Data. Curr Protoc Bioinformatics. 2016;56:15 0 1- 0 8. doi: 10.1002/cpbi.20.
9. Raine KM, Hinton J, Butler AP, Teague JW, Davies H, Tarpey P, et al. cgpPindel: Identifying Somatically Acquired Insertion and Deletion Events from Paired End Sequencing. Curr Protoc Bioinformatics. 2015;52:15 7 1-2. doi: 10.1002/0471250953.bi1507s52.
10. Shen R, Seshan VE. FACETS: allele-specific copy number and clonal heterogeneity analysis tool for high-throughput DNA sequencing. Nucleic Acids Res. 2016;44(16):e131. doi: 10.1093/nar/gkw520.
